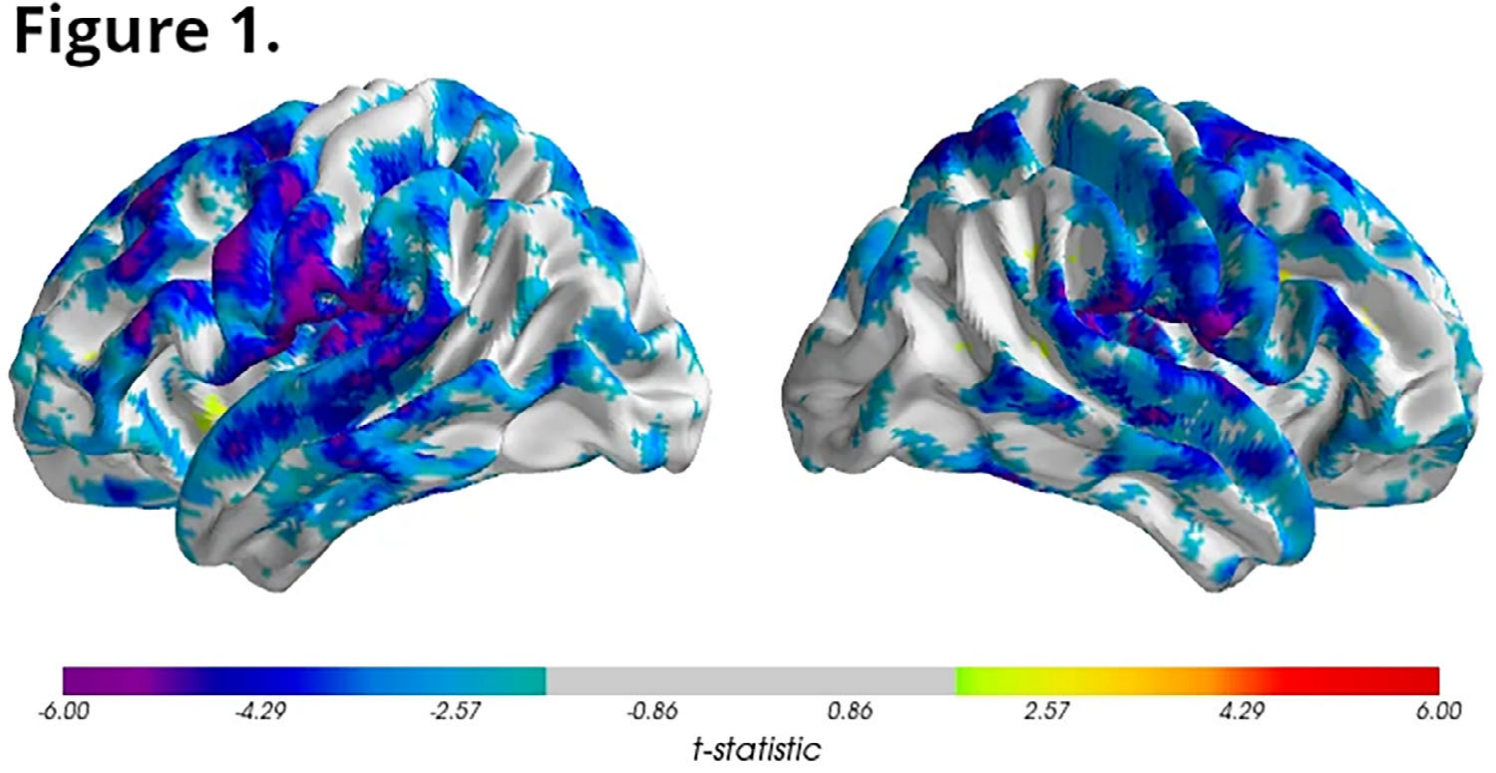# Advanced MRI biomarkers for efficient detection and monitoring of Corticobasal Degeneration (CBD) progression in clinical trials

**DOI:** 10.1002/alz70862_109830

**Published:** 2025-12-23

**Authors:** Simone P Zehntner, Jean‐Philippe Coutu, Felix Carbonell, Alex P Zijdenbos, Barry J Bedell

**Affiliations:** ^1^ Biospective Inc., Montreal, QC Canada; ^2^ Biospective Inc, Montreal, QC Canada

## Abstract

**Background:**

Corticobasal Degeneration (CBD) is a rare neurodegenerative disorder marked by misfolded tau protein accumulation, leading to neuronal degeneration. CBD shares clinical features with other Parkinsonian syndromes, complicating accurate diagnosis. MRI biomarkers, such as volumetric and diffusion MRI (dMRI) analyses, are essential for understanding disease progression and enhancing clinical trial efficiency. This study incorporates advanced imaging techniques to explore the progression of CBD and its differentiation from similar disorders.

**Methods:**

MRI data from CBD, PSP, and healthy controls were sourced from the 4‐Repeat Tauopathy Neuroimaging Initiative (4RTNI) and Frontotemporal Lobar Degeneration Neuroimaging Initiative (FTLDNI). Fully‐automated image processing was performed using the PIANO™ software platform for gray matter density and regional volume assessments. dMRI metrics, such as radial diffusivity (RD) and free water (FW), were analyzed to track microstructural changes. Sample size calculations were conducted to estimate the number of participants required to detect therapeutic effects in a clinical trial.

**Results:**

CBD subjects showed rapid brain atrophy with notable decreases in gray matter density. Figure 1 illustrates the surface projections of the statistically significant (FDR‐corrected, q=0.05) gray matter changes over a 12‐month period within the CBD population, particularly in the sensorimotor, parietal, and temporal cortices. Volumetric analysis revealed 2.5%‐5% reductions in these regions, with subcortical atrophy noted in the thalamus and hippocampus. Sample size estimates indicated that 30‐44 subjects per arm are required to detect a 60% reduction in atrophy. dMRI metrics show up to 5% change in regional mean diffusivity in white matter and cortical regions (sensory, motor, frontal, and parietal) over the same 12‐month period. Sample size estimates for diffusion metrics as similar to those for the atrophy assessments. Compared to other tools, such as FreeSurfer, the PIANO™ platform significantly reduced sample size requirements while maintaining sensitivity.

**Conclusion:**

Advanced MRI methodologies, combining volumetric and diffusion‐based analyses, enable precise tracking of CBD progression and differentiation from PSP. This approach minimizes sample size requirements, can potentially accelerate clinical trials, and facilitates early evaluation of disease‐modifying therapies. The integration of robust imaging biomarkers into clinical trials is critical for improving diagnostic accuracy and therapeutic interventions for rare neurodegenerative diseases, like CBD.